# Long-term participation in community-based group resistance exercises delays the transition from robustness to frailty in older adults: a retrospective cohort study

**DOI:** 10.1186/s12199-021-01028-x

**Published:** 2021-10-20

**Authors:** Chisato Hayashi, Hiromitsu Toyoda, Soshiro Ogata, Tadashi Okano, Sonoe Mashino

**Affiliations:** 1 Research Institute of Nursing Care for People and Community, University of Hyogo, 13-71 Kitaoji-cho, Akashi, Hyogo 673-8588 Japan; 2 Department of Orthopaedic Surgery, Osaka City University Graduate School of Medicine, 1-4-3 Asahi-machi, Abeno-ku, Osaka-City, Osaka 545-8585 Japan; 3 Department of Preventive Medicine and Epidemiology, National Cerebral and Cardiovascular Center, 6-1 Kishibeshinmachi, Suita, Osaka, 564-8565 Japan

**Keywords:** Lively 100-years-old physical exercise, Community-dwelling older adults, Frailty, Kihon Checklist

## Abstract

**Background:**

How community-based group resistance exercises affect the transition from robustness to frailty remains unclear. Thus, we conducted a retrospective cohort study to determine whether the trajectory from robustness to frailty over age differed depending on the duration of participation in group exercises.

**Methods:**

We analyzed the Kihon Checklist (KCL) score of community-dwelling elderly residents of Sumoto city, Hyogo prefecture, who participated in community-based group resistance exercises between April 2010 and December 2019. Finally, 2567 older individuals were analyzed using multilevel modeling. The explanatory variables of interest were the frailty score measured using the KCL for each individual, where 0–3, 4–7, and ≥8 points denoted robustness, pre-frailty, and frailty, respectively. We considered age, sex, systolic blood pressure, pulse, duration of participation, and change in KCL score from baseline as possible confounders. Participants were classified as follows based on the duration of participation in the exercises: <3 times, short-term participation group; 4–6 times; mid-term participation group; and 7–13 times, long-term participation group. The mean duration from the baseline physical test for the total sample was 2.35 years (SD=2.51).

**Results:**

The participants’ mean total KCL score at baseline was 4.9±3.7. Multilevel modeling analysis revealed that the KCL scores changed by 0.82 points for each additional year of age (*p*<0.001) and changed by − 0.93 points for long-term participate group (*p*<0.001). The Estimated Marginal Means (EMM) of the KCL score was 3.98 (95%CI: 3.69, 4.28) points in the short-term participation group and was significantly worse than that of the long-term participation group at 70 years of age (*p*=0.001). The EMM was 4.49 (95%CI: 4.24, 4.74) at 75 years of age in the mid-term participation group and was significantly worse than that of the long-term participation group. The EMM was 3.87 (95%CI: 3.57, 4.16) in the long-term participation group and significantly better than that of the short-term (*p*<0.001) and mid-term (*p*=0.002) participation groups.

**Conclusion:**

Participation in community-based group resistance exercises prolongs the transition from robustness to frailty. The improved KCL scores at baseline in the long-term participation group remained in the robust range at 75 years of age, which suggests the importance of initiating participation before the onset of functional decline.

**Supplementary Information:**

The online version contains supplementary material available at 10.1186/s12199-021-01028-x.

## Background

Frailty is an age-related decrease in the physiological reserve characterized by a weakened response to stressors and an increased risk of poor clinical outcomes [1, 2]. It is considered a dynamic concept with variable severity, especially at the individual level [3]. Pre-frailty is a condition that occurs before the onset of frailty, which is more likely to revert to the robust state compared to frailty; thus, it is important to implement interventions in the pre-frailty phase. Kitamura et al. (2020) found that the contribution of frailty and pre-frailty in the early stages of old age (65–74 years of age) to mortality and the requirement of care was higher than that of other lifestyle-related diseases [4]. A recent systematic review and meta-analysis conducted in Japan reported that the estimated prevalence of frailty and pre-frailty was 7.4 % and 48.1%, respectively [5].

Although pre-frailty is highly prevalent in community-dwellers, the standard definition and assessment tools for this condition are lacking. Sezgin et al. (2020) proposed a comprehensive definition that described pre-frailty as “a multi-dimensional concept, an early and reversible risk-state before frailty, which is defined operationally by existing frailty screening and assessment tools” [6]. A recent study found no age-based differences between frailty and pre-frailty [7]. Most individuals who participate in these resident-led exercises are older adults who live independently, and their condition varies from robustness to frailty. The Kihon Checklist (KCL), which was developed to identify individuals eligible for long-term care prevention projects, has been used to screen older people who are likely to require long-term care in the near future in Japan. This checklist consists of 25 items, including instrumental activities of daily living, physical function, nutritional status, oral function, cognitive function, confinement, and depression (Ministry of Health, Labor and Welfare, 2006). Total scores exceeding 8/25 are considered to denote the state of frailty. Total scores ranging between 4/25 and 7/25 are considered to denote the state of pre-frailty [8].

The resident-led community group resistance exercise called “lively 100-years-old physical exercise” [9] intended for all ages is being implemented for care prevention in Sumoto City, Hyogo, Japan. It recommended that community residents should gather proactively and designate a place for social interaction in addition to muscle-strengthening exercises. Resistance training is beneficial for enhancing muscle strength, walking speed, heart disease, fall prevention, cognitive function, and self-esteem [10]. Participation in the “lively 100-years-old physical exercise” has been shown to positively impact lower extremity muscle strength, walking speed, and motor fitness scale scores [11].

Evidence obtained from a systematic review suggests that physical exercise programs are generally effective for reducing frailty only when conducted in groups [2]. Although numerous studies have investigated frailty in older individuals, little is known about the manner in which community-based group resistance exercises affect the transition from robustness to frailty. This study aimed to estimate the KCL score in community-dwelling individuals who participated in group resistance exercises using multilevel modeling (MLM), which can model the longitudinal changes from the state of robustness to that of frailty.

## Methods

### Study design and participants

Our data were derived from physical tests conducted between April 2010 and December 2019 who participated in the community-based resistance exercise program called the “lively 100-years-old physical exercise.” This study incorporated a retrospective cohort design. The data were used from those held by the Care and Welfare Division in Sumoto City, Hyogo prefecture. The physical test registry who took at least one physical test included a total of 2570 participants. However, gender was unknown in the data of 3 participants. Finally, a total of 2567 older adults' KCL scores were analyzed in this study.

We analyzed whether the trajectories of the KCL scores of community-dwelling adults who participated in the “lively 100-years-old physical exercise” differed from those of individuals who did not continuously participate in this regimen. The estimated KCL score at each age of the three groups (short-, middle- and long-term participation) were calculated, along with criteria for the age-based models for the transition from robustness to frailty.

This study was approved by the Ethics Committee of the University of Hyogo (No. 2019F21).

### Data

#### Exposure

The regimen comprised exercises with weights attached to the limbs. Strength training included the following 7 types of muscle-strengthening exercises: lifting both arms up, lifting both arms to the side, getting up from the chair, knee extension exercises, knee lift exercises, lateral leg lifting exercises, and standing hip extension exercises [9]. The exercise sessions were conducted once a week.

#### Outcomes

We assessed frailty using the KCL score. The KCL consists of 25 easy-to-answer questions that assess 7 subcategories: instrumental activities of daily living, oral function, physical function, cognitive function, depression, nutrition, and house-boundness. Satake et al. (2016) verified that an individual’s total KCL score is closely correlated with the number of Fried’s phenotypes and proposed that KCL scores of ≥4 should be defined as pre-frailty (sensitivity, 70.3%; specificity, 78.3%) and scores of ≥8 should be defined as frailty (sensitivity 89.5%; specificity 80.7%) [8].

#### Covariates

We controlled four variables such as age (years), sex, systolic blood pressure, and pulse rate.

#### Duration of exercises

The older adults underwent physical fitness tests every 4 months in the first year after starting the program, and thereafter, once annually, as per the “lively 100-years-old exercise” regimen. Older adults who participated in the “lively 100-years-old exercise” were required to answer the KCL at physical fitness tests. The KCL scores were derived from physical tests conducted between April 2010 and December 2019; some older adults stopped participating and then started again. If the duration of participation is defined as the time from baseline to participation in the last physical fitness test, it will be longer for participants who stopped once in between.

Therefore, all participants in this analysis were divided into three groups according to the total number of enrolled and completed physical test sessions. The intervals between the physical fitness tests are standardized, as mentioned above. The total number of physical fitness tests per participant ranged from 1 to 13, of which 3 tests lay in the 25th percentile, and 7 tests lay in the 75th percentile. Participants who underwent the tests <3 times were placed in the short-term participation group, those who underwent the tests 4 to 6 times were placed in the mid-term participation group, and those who underwent the tests 7 to 13 times were placed in the long-term participation group [11]. As a result, a mean number of physical tests were 1.7 in the short-term participation group, 4.8 in the mid-term participation group, and 8.2 in the long-term participation group (Table 1). Furthermore, the duration of observation was 0.8 years in the short-term participation group, 3.8 years in the mid-term participation group, and 6.4 years in the long-term participation group (Table 1).

**Table 1:** Baseline characteristics of all participants and each group

	All	Short-term	Mid-term	Long-term	
	***n*** = 2567	***n*** = 1553	***n*** = 635	***n*** = 379	
	Mean (SD)	Mean (SD)	Mean (SD)	Mean (SD)	***P*** value
Times of physical test (times)	3.4 (2.5)	1.7 (0.8)	4.8 (0.8)	8.2 (1.2)	<0.001
Duration of observation (years)	2.4 (2.5)	0.8 (1.2)	3.8 (1.6)	6.4 (1.2)	<0.001
Age (years old)	74.2 (8.0)	74.4 (8.5)	74.2 (7.2)	73.6 (6.7)	0.133
Sex					
Male (people)	434 (16.9%)	276 (17.8%)	95 (15.0%)	63 (16.6%)	
Female (people)	2133 (83.1%)	1277 (82.2%)	540 (85.0%)	316 (83.4%)	0.275
Systolic blood pressure (mmHg)	140.6 (19.5)	140.3 (19.3)	140.7 (19.8)	141.1 (19.9)	0.773
Pulse rate (times/minute)	78.1 (12.0)	78.1 (12.2)	78.0 (11.4)	78.0 (12.0)	0.979
Subscore of KCL					
IADL (0–5)	3.8 (2.8)	3.9 (2.9)	3.6 (2.5)	3.3 (2.3)	<0.001
Oral function (0–3)	0.8 (0.9)	0.8 (0.9)	0.8 (0.9)	0.8 (0.9)	0.308
Physical function (0–5)	1.4 (1.2)	1.5 (1.3)	1.4 (1.2)	1.3 (1.2)	0.043
Cognitive function (0–3)	0.7 (0.8)	0.7 (0.8)	0.6 (0.7)	0.6 (0.8)	0.159
Depression (0–5)	1.1 (1.4)	1.1 (1.4)	1.0 (1.3)	1.0 (1.4)	0.057
Nutrition (0–2)	0.3 (0.5)	0.3 (0.5)	0.3 (0.5)	0.2 (0.4)	0.017
Houseboundness (0–2)	0.3 (0.5)	0.3 (0.5)	0.3 (0.5)	0.2 (0.4)	0.013
Total KCL score (0–25)	4.9 (3.7)	5.1 (3.8)	4.6 (3.4)	4.2 (3.3)	<0.001

*KCL* Kihon Checklist, *IADL* instrumental activities of daily living

### Statistical analyses

A model in which the effects of entities that are regarded as fixed values in the population (fixed effect) are mixed with the effects of entities that behave stochastically in the same population (random effect) is called a mixed model. The multilevel model is a type of mixed model. The fixed effect is the parameter estimated by the model that is obtained as a constant, such as the intercept and the regression coefficient in regression analysis. Conversely, the random effect is one in which the effect varies stochastically, and the parameters vary for each subject.

We used the linear mixed-effects model to identify the difference in the slope of frailty [12]. Since the measurements within the same subject are correlated, it is necessary to account for the variance-covariance structure. In this study, the temporal variable (age) was regarded as a continuous scale in the model, the correlations between the observed values for the same individual were allowed, and the variable that distinguished the individuals was the variable effect. This enabled us to test the magnitude of changes over time and determine the correlations between intercepts and slopes and differences in the intercepts and slopes based on the differences in the characteristics of the individuals. We assumed that the random effects distribution was normal and extended to estimate model parameters with the maximum likelihood methods. The model was used to estimate the mean at each point in the reference grid for each age from 65 to 90 years. Marginal means were estimated as equally weighted means of these predictions at specified margins.

We estimated the KCL scores on a random intercept and random slope for each participant of the community-based group resistance exercise. In model 1, we estimated the age effect as the interaction effect between age, the indicator variable (duration of participation), and the change in the KCL score from baseline. Moreover, we estimated the effect of sex, systolic blood pressure, and pulse rate as confounders. The KCL scores collected at 13-time points were analyzed using mixed-effects models with random intercepts and slopes over time. We derived regression coefficients, 95% confidence interval (95% CI), and *p* values from the models.

All statistical analyses were conducted using R version 4.1.0 (Vienna, Austria) [13], and the lme4 package [14] was used to fit the mixed-effects models [15]. The Match lt package [16] and Zeling [17] package were used for sensitivity analyses. Tukey’s post hoc test was used to compare the data from different groups. The level of significance was set at *p* < 0.05.

## Results

### Baseline demographic characteristics

The mean duration from the baseline physical test for the entire sample was 2.35 years (SD = 2.50), while that for the short-term, mid-term, and long-term participation groups was 0.75 years (SD = 1.21), 3.81 years (SD = 1.60), and 6.44 years (SD = 1.19), respectively.

The participants’ baseline demographic characteristics are presented in Table 1. The total number of participants was 2567. The participants’ mean total baseline KCL score was 4.9±3.7 points (mean age: 74.2 years). The mean total baseline KCL score was 5.1±3.8 points for the short-term participation group (mean age: 74.4 years), 4.6±3.4 points for the mid-term participation group (mean age: 74.2 years), and 4.2±3.3 points for the long-term participation group (mean age: 73.6 years). The difference in the mean score of the groups was significant at baseline (*p*< 0.001). Moreover, the difference in the mean age of the groups was not significant at baseline (*p* = 0.133). The proportion of men in the sample was 16.9%.

The participants’ mean baseline instrumental activities of daily living (IADL) subscore of KCL was 3.9±2.9 points for the short-term participation group, 3.6±2.5 points for the mid-term participation group, and 3.3±2.3 points for the long-term participation group. The difference in the mean IADL subscore of the groups was significant at baseline (*p*< 0.001).

### Multilevel linear regression models

**Fig. 1:**
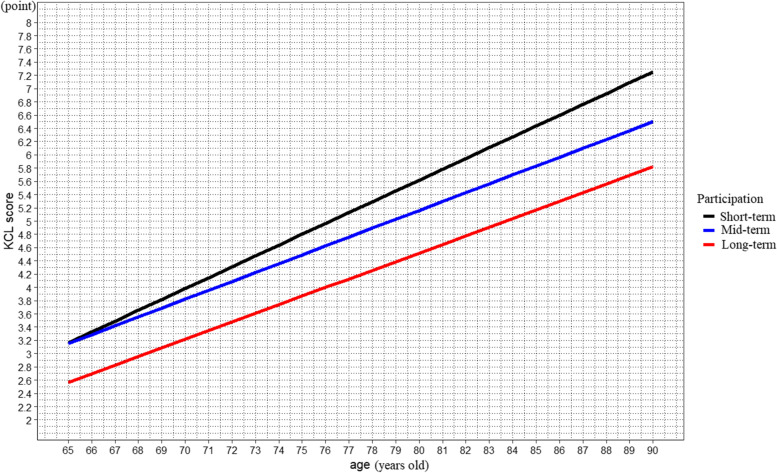
Slopes of the changes and estimated marginal means (95% confidence interval) of the KCL scores of participants who performed community-dwelling group exercise. The slopes and estimated marginal means were based on results described in Table 2, obtained from the linear mixed models. The bar lines represent 95% confidence intervals for the estimated marginal means at each 5-year-age point. KCL, Kihon Checklist

We specifically aimed to determine if the trajectory from robustness to frailty over age differed for the short-term, mid-term, and long-term participation groups. This was achieved by assessing the fixed-effects interaction between age and the groups. Figure 1 shows the estimated KCL scores of older adults participating in community-based resistance exercise at each age. The short-term participation group reached the pre-frailty range at 71 years of age, the mid-term participation group at 72 years of age, and the long-term participation group at 76 years of age (Appendix Table 1). The KCL scores increased throughout the follow-up period in older adults in all groups who participated in community-based group resistance exercises in this investigation of the transition from robustness to pre-frailty.

For sensitivity analyses, we divided the participants into two groups with the same KCL score at baseline and different durations of exercise participation and compared the two groups. We divided the participants into the long- and short-term participation groups by duration of participation (divided by median value; 5 times). The baseline KCL score was matched by using paired matching to select individuals’ KCL scores, which were the same at baseline. The sample sizes were 1699 for the short-term participation group and 346 for the long-term participation group after matching. The covariates were duration of participation and age after centering mean age (75 years old). As a result, the estimates before and after matching were almost identical. The estimated KCL scores were 5.11 (standard adjusted analysis, 95%CI: 4.95, 5.27) and 5.00 (matched analysis, 95%CI: 4.84, 5.16). For the long-term participation group, the KCL scores changed by − 0.57 (95%CI: − 0.96, − 0.17) points by standard adjusted analysis and by − 0.48 (95%CI: − 0.87, − 0.10) points by matched analysis.

The estimated marginal means are presented in Table 2. The estimated KCL score was 3.98 (95%CI: 3.69, 4.28) points in the short-term participation group, reached the pre-frailty range, and was significantly worse than that of the long-term participation group at 70 years of age (*p*=0.001). The estimated score was in the robust range at 70 years of age in both the mid- and long-term participation groups. The estimated KCL score was 4.49 (95%CI: 4.24, 4.74) at 75 years of age in the mid-term participation group and was significantly worse than that of the long-term participation group. At 75 years of age, the scores of the short- and mid-term participation group belonged to the pre-frailty range. The estimated score was 3.87 (95%CI: 3.57, 4.16) in the long-term participation group, which was in the robust range and significantly better than that of the short-term (*p*<0.001) and mid-term (*p*=0.002) participation groups. At 80 years of age, the scores of all participating groups belonged to the pre-frailty range. At 90 years of age, the estimated KCL score for older adults participating in community-based group resistance exercises was 7.25 (95%CI: 6.64, 7.87) in the short-term participation group, 6.50 (95%CI: 5.96, 7.04), and 5.82 (95%CI: 5.25, 6.39). The score was over the pre-frailty range at over 80 years of age; however, the score did not reach the frailty range threshold at 90 years of age in all groups.

**Table 2:**
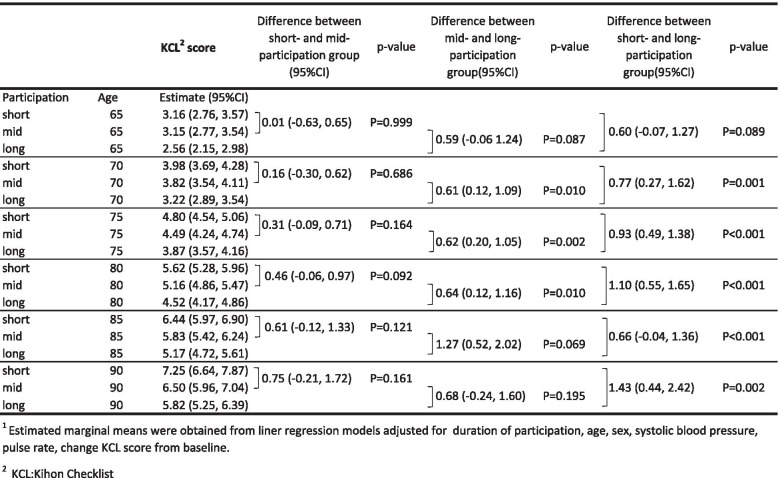
Estimated marginal means (95%CI)1 of KCL2 score obtained by linear mixed models

^1^Estimated marginal means were obtained from linear regression models adjusted for duration of participation, age, sex, systolic blood pressure, pulse rate, and change KCL score

^2^*KCL* Kihon Checklist

Table 3 presents the regression coefficients obtained from the multilevel linear regression models. Age was centered using the mean age (75 years) of all participants. Model 1 was an unadjusted model. One way to judge a model is to compare the estimated means with the observed means to ascertain the accuracy with which they are represented by the model. This excellent fit of the estimated marginal means to the observed data supports the hypothesis that the change across age is linear. We controlled for the duration of participation, change in KCL score from baseline, sex, systolic blood pressure, and pulse rate in model 2. The KCL scores changed by 0.82 points for each additional year of age, which was statistically significant (*p*<0.001). The KCL scores changed by − 0.93 points for long-term participate group, which was statistically significant (*p*<0.001). Change in KCL score from baseline was statistically significant (*p*<0.001), and the estimate was changed by 0.51 points for each additional KCL score.

**Table 3:** Regression coefficients obtained from the multilevel linear regression models

	KCL^**5**^ score Mean difference of Kihon Checklist score by 1-unit increase of each explanatory variable	
	*N*=2414 (total^1^=7928)	*p* value
**Model 1**^2^		
Intercept	4.61 (4.49, 4.73)	*P*<0.001
Age^4^ (years old)	0.75 (0.67, 0.82)	*P*<0.001
**Model 2**^3^		
Intercept	3.90 (3.33, 4.46)	*P*<0.001
Age^4^ (years old)	0.82 (0.64, 0.99)	*P*<0.001
Participation		
Short	0 (Reference)	
Mid	− 0.31 (− 0.64, 0.02)	*P*=0.069
Long	− 0.93 (− 1.30, − 0.56)	*P*<0.001
Change of KCL score from baseline	0.51 (0.49, 0.52)	*P*<0.001
Interaction terms between age and participation		
Age:short	0 (Reference)	
Age:mid	− 0.15 (− 0.38, 0.08)	P=0.211
Age:long	− 0.17 (− 0.40, 0.07)	P=0.162

^1^Records due to repeated physical test

^2^Model 1 was unadjusted

^3^Model 2 was adjusted for sex, systolic blood pressure, and pulse rate

^4^Age was centered by 75 years old

^5^KCL was Kihon Checklist

## Discussion

To the best of our knowledge, this is the first study to determine the relationship between longer participation in group resistance exercises and its substantially positive effect in preventing frailty. Notably, this is the first MLM analysis to be applied to the KCL scores of participants who performed the lively “100-years-old physical exercise program.” Not only did this study find that the KCL scores declined with age, but also that there are differences in the slope depending on the duration of participation in group exercises in the community.

The study found that the estimated KCL scores of older adults participating in community-based resistance exercise lay in the robust range in all groups at 75 years of age and lay within the pre-frailty range in all groups at 90 years of age. However, the short-term participation group reached the pre-frailty range at 71 years of age, the mid-term participation group at 72 years of age, and the long-term participation group at 76 years of age. The robust state was prolonged by 5 years in the long-term participation group compared to the short-term participation group.

Since the study population included older people participating in community-based group resistance exercises, several were considered to have maintained the pre-frailty level. This coincides with the findings of earlier studies [18]. The presence of factors that promote frailty, such as bereavement due to the death of a family member, surgery due to illness, or hospitalization, may accelerate the progression to frailty. Older individuals who have experienced such events may require support to ensure their continued participation in these sessions.

Multilevel modeling analysis revealed that the KCL scores changed by 0.82 points for each additional year of age. Furthermore, the improved KCL scores at baseline in the long-term participation group remained in the robust range at 75 years of age, suggesting the importance of initiating participation before the onset of functional decline. Of the 42,775 community-dwelling older adults from 16 studies (mean follow-up duration: 3.9 years), the condition of 23.1% of individuals with pre-frailty improved to the robust state, compared to only 3.3% of those who were in the state of frailty at baseline [19]. A recent study found that participation in exercise-based social activities is associated with the reversal of frailty progression; however, the two-stage improvement from frailty to robustness was observed in only 5.8% of the population [20]. This suggests that it is important to maintain the prefrailty state for a long time and to prevent the deterioration of the frail state.

In this study, community-based resistance group exercise prevented frailty and hampered the rate of deterioration with age. Previous research reported that resistance training increased strength and gait speed in frail adults [21]. Another study found that gait speed could be a useful indicator to classify older people at high risk of requiring long-term care [22]. Community-based initiatives where older people proactively gather together and exercise in their neighborhoods have been shown to delay functional decline associated with aging, which should motivate the government, older individuals, and their families to work on long-term care prevention. Seino et al. concluded that the decision to exercise alone or with others might be unimportant when aiming to maintain physical function; however, exercising with others may further enhance physical activity [23]. Kanamori et al. found that a higher frequency of exercise with others has important health benefits, irrespective of the total frequency of exercise. Exercise can influence the muscles during aging, and their role as part of a lifestyle essential to healthy aging should be emphasized [24].

A recent study showed that the number of social participation activities, such as sports clubs or sports groups, was significantly associated with lower odds of prefrailty [25]. Moreover, a systematic review reported that club- or team-based sports seemed to be associated with improved health outcomes compared to individual activities due to their social nature [26]. As it has been reported that friendship networks served as good predictors of all-cause mortality in the elderly Japanese population [27], community-based exercise can be expected to be effective as a form of physical activity and as a place for forming friendships. Incorporating lifelong club and team sports into people's lives may help prevent physical and social frailty.

This study had three limitations. First, we could not determine other potentially related factors, such as the history of diseases, because this study used secondary data. Second, we could not assess the participants’ final physical tests as the reasons for quitting the exercise group were unknown. Third, we are unable to determine the causal relationship as this retrospective cohort study used secondary data.

In conclusion, participation in community-based group resistance exercises has a positive effect on the prevention of frailty in the older adults, despite the effects of aging. Multilevel modeling analysis revealed that the KCL scores changed by 0.82 points for each additional year of age, which was statistically significant (p<0.001). The KCL scores changed by − 0.93 points for long-term participate group, which was statistically significant (*p*<0.001). The EMM of KCL scores for older adults participating in community-based group resistance exercise did not reach the frailty threshold at the age of 90 years. The improved KCL scores at baseline in the long-term participation group remained in the robust range at 75 years of age, which suggests the importance of initiating participation before the onset of functional decline. We hope that our findings can lay the basis for further studies in this field.

## Supplementary Information

**Additional file 1: Appendix Table 1**. Estimated marginal means (95%CI)1 of KCL2 score obtained by linear mixed models at each age.

## Data Availability

The data that support the findings of this study are available from the corresponding author, but restrictions apply to the availability of these data, which were used under license for the current study, and so are not publicly available. However, data are available from the authors upon reasonable request and with permission of the Ethics Committee of the University of Hyogo. All authors had full access to the study data in the research room of CH, University of Hyogo.

## Abbreviations

KCL: Kihon Checklist
MME: Estimated marginal means
IADL: Instrumental activities of daily living
