# Supplementary material for: Long-term participation in community-based group resistance exercises delays the transition from robustness to frailty in older adults: a retrospective cohort study
Source: Environ Health Prev Med. 2021 Oct 20;26:105. doi: 10.1186/s12199-021-01028-x (PMC8529757; doi:10.1186/s12199-021-01028-x)
Supplement: Supplementary file 1 — Additional file 1: Appendix Table 1. Estimated marginal means (95%CI)1 of KCL2 score obtained by linear mixed models at each age. [file 12199_2021_1028_MOESM1_ESM.docx]

| Appendix Table 1. Estimated marginal means (95%CI)^1^ of KCL^2^ score obtained by linear mixed models at each age. | | | | | |
| --- | --- | --- | --- | --- | --- |
|  |  |  | Participation |  |  |
|  | short-term |  | mid-term |  | long-term |
|  | KCL^2^ score | | | | |
| Age | Estimate (95%CI) |  | Estimate (95%CI) |  | Estimate (95%CI) |
| 65 | 3.16 (2.76, 3.57) |  | 3.15 (2.77, 3.54) |  | 2.56 (2.15, 2.98) |
| 66 | 3.33 (2.95, 3.71) |  | 3.29 (2.93, 3.65) |  | 2.69 (2.30, 3.09) |
| 67 | 3.49 (3.14, 3.84) |  | 3.42 (3.08, 3.76) |  | 2.82 (2.45, 3.20) |
| 68 | 3.66 (3.32, 3.99) |  | 3.55 (3.23, 3.87) |  | 2.95 (2.60, 3.31) |
| 69 | 3.82 (3.51, 4.13) |  | 3.69 (3.39, 3.99) |  | 3.08 (2.75, 3.42) |
| 70 | 3.98 (3.69, 4.28) |  | 3.82 (3.54, 4.11) |  | 3.22 (2.89, 3.54) |
| 71 | 4.15 (3.87, 4.42) |  | 3.96 (3.68, 4.23) |  | 3.35 (3.03, 3.66) |
| 72 | 4.31 (4.04, 4.58) |  | 4.09 (3.83, 4.35) |  | 3.48 (3.17, 3.78) |
| 73 | 4.47 (4.21, 4.74) |  | 4.22 (3.97, 4.48) |  | 3.61 (3.31, 3.91) |
| 74 | 4.64 (4.38, 4.90) |  | 4.36 (4.11, 4.61) |  | 3.74 (3.44, 4.03) |
| 75 | 4.80 (4.54, 5.06) |  | 4.49 (4.24, 4.74) |  | 3.87 (3.57, 4.16) |
| 76 | 4.96 (4.69, 5.24) |  | 4.63 (4.37, 4.88) |  | 4.00 (3.70, 4.30) |
| 77 | 5.13 (4.85, 5.41) |  | 4.76 (4.50, 5.02) |  | 4.13 (3.82, 4.43) |
| 78 | 5.29 (4.99, 5.59) |  | 4.89 (4.62, 5.17) |  | 4.26 (3.94, 4.58) |
| 79 | 5.45 (5.14, 5.77) |  | 5.03 (4.74, 5.31) |  | 4.39 (4.06, 4.72) |
| 80 | 5.62 (5.28, 5.96) |  | 5.16 (4.86, 5.47) |  | 4.52 (4.17, 4.86) |
| 81 | 5.78 (5.42, 6.14) |  | 5.30 (4.97, 5.62) |  | 4.65 (4.29, 5.01) |
| 82 | 5.95 (5.56, 6.33) |  | 5.43 (5.09, 5.77) |  | 4.78 (4.40, 5.16) |
| 83 | 6.11 (5.70, 6.52) |  | 5.56 (5.20, 5.93) |  | 4.91 (4.51, 5.31) |
| 84 | 6.27 (5.83, 6.71) |  | 5.70 (5.31, 6.08) |  | 5.04 (4.62, 5.46) |
| 85 | 6.44 (5.97, 6.90) |  | 5.83 (5.42, 6.24) |  | 5.17 (4.72, 5.61) |
| 86 | 6.60 (6.10, 7.10) |  | 5.96 (5.53, 6.40) |  | 5.30 (4.83, 5.77) |
| 87 | 6.76 (6.24, 7.29) |  | 6.10 (5.64, 6.56) |  | 5.43 (4.94, 5.92) |
| 88 | 6.93 (6.37, 7.48) |  | 6.23 (5.74, 6.72) |  | 5.56 (5.04, 6.08) |
| 89 | 7.09 (6.50, 7.68) |  | 6.37 (5.85, 6.88) |  | 5.69 (5.15, 6.24) |
| 90 | 7.25 (6.64, 7.87) |  | 6.50 (5.96, 7.04) |  | 5.82 (5.25, 6.39) |
| ^1^ Estimated marginal means were obtained from liner regression models adjusted for frequency of participation, age, sex, systolic blood pressure, pulse rate, change KCL score from baseline. | | | | | |
| ^2^  KCL:Kihon Checklist |  |  |  |  |  |
